# Supporting unpaid carers during section 17 leave from mental health in-patient wards: carer and practitioner perspectives

**DOI:** 10.1192/bjo.2025.16

**Published:** 2025-03-26

**Authors:** Laura Tucker, Nicola Moran, Ruth Naughton-Doe, Emma Wakeman, Mark Wilberforce, Martin Webber

**Affiliations:** School for Business and Society, University of York, York, UK; Independent Social Worker, UK

**Keywords:** Mental health, unpaid carers, section 17 leave, hospital leave, carer support

## Abstract

**Background:**

Care planning for recovery and to work towards hospital discharge is integral to good practice in mental health in-patient settings. Authorised leave from hospital, especially for those who are detained, can be used to check readiness for discharge and to maintain social connections that support a patient’s recovery journey. Leave therefore often involves friends and family, or ‘carers’. However, carer involvement in planning leave is limited, and carers struggle with feeling unsupported during the leave.

**Aims:**

This study aimed to explore carers’ and mental health practitioners’ subjective experiences of leave in the context of implementing a set of practice guidelines for involving carers in planning and undertaking leave from hospital.

**Method:**

Nine wards in six National Health Service trusts were recruited to implement the guidelines. Interviews were undertaken with carers (*n* = 6) and practitioners (*n* = 3) from these implementation wards and with carers (*n* = 7) from nine usual care wards. A further ten practitioners completed an anonymous online survey. Data were analysed thematically.

**Results:**

Carers’ experiences on both implementation and usual care wards indicated variable levels of involvement, with carers positioned as partners in care, observers of care or outsiders to care. Practitioner perspectives highlighted practical, structural and conceptual challenges in working with carers, which precluded effective implementation of the guidelines.

**Conclusions:**

The guidelines reflected what both carers and practitioners described as good practice, but resource limitations, unclear responsibilities and perceptions of carer roles limited engagement. Implementing approaches to working with carers in in-patient settings requires resourcing and clear role definition within staff–carer relationships.

## Unpaid care and section 17 leave from hospital

An estimated 1.5 million people in the UK care for a friend or family member experiencing mental health difficulties,^1^ a number anticipated to rise alongside mental health need in the aftermath of the coronavirus pandemic^2,3^ and the cost of living crisis.^4^ Where mental health deterioration requires detention in hospital, linked to worsening of mental health with a corresponding increased risk to self or others, engaging carers in patients’ treatment is beneficial for both the carer and the patient.^5,6^ However, carers report that it is more difficult for them to access support in mental health than in other services, with an increased likelihood that services will not meet their needs.^7^ Systematic reviews of the evidence similarly indicate that carers tend to be unsupported or uninvolved in treatment planning.^8–10^

Good practice in in-patient contexts recommends early planning for discharge and carer involvement.^11,12^ A central element of the plan for detained patients can involve authorised leave from hospital under section 17 of the Mental Health Act (MHA) 1983 (s.17 leave). S.17 leave can test readiness for discharge and help maintain connections with friends and family, which can contribute to recovery.^13,14^ However, carer involvement in decision-making around s.17 leave can be limited,^15^ with the experience of leave negatively affecting carers’ well-being in the context of limited support from in-patient settings.^16^

## The s.17 standard for carers

**Fig. 1:**
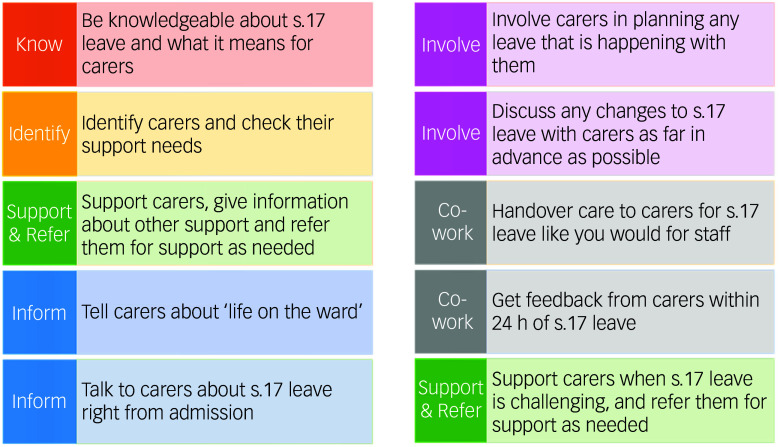
The section 17 (s.17) standard for carers.

An earlier phase of the study reported in this paper explored carers’ and practitioners’ views on how carers could be better involved and supported by in-patient services around s.17 leave.^17^ This identified three key themes: challenges of carer support; communication and co-working between staff and carers; and the need for clear procedures. Practitioners experience challenges in working confidently with carers^15,18,19^ and have concerns around breaching confidentiality.^1,15,20^ They can also work within practice environments that are not supportive of working with carers^15^ or are too resource limited to enable this.^1^ Acknowledging this, the themes were translated into an actionable approach through a ten-point ‘s.17 standard’ (Fig. 1) to inform how in-patient practitioners could work with carers to plan and undertake s.17 leave.

This study aimed to explore carers’ and practitioners’ experiences of leave in the context of implementing the s.17 standard and whether this differed from experiences in usual practice.

## Method

### Design

The study adopted an interpretivist qualitative design to explore practitioners’ and carers’ subjective experiences of leave and the implementation of the s.17 standard. Semi-structured interviews were used to gather data from practitioners and carers, with data also collected from practitioners using an anonymous online self-completed survey.

### Setting

The study was conducted in nine wards that implemented the s.17 standard (‘implementation wards’), and nine wards that continued ‘business as usual’ (‘usual care wards’), across six National Health Service (NHS) mental health trusts in England, providing population-wide mental health care and representing a diverse mix of urban and rural contexts^21^ between March 2022 and May 2023.

### Eligibility

Adult carers were eligible to take part if they were providing unpaid care to adult in-patients detained under MHA s.2 for assessment or s.3 for treatment in either implementation or usual care wards who had not yet been given s.17 leave from the ward. All practitioners working on implementation wards were eligible to participate.

### Participant recruitment

All known eligible carers and all implementation ward staff were provided with recruitment information and consent forms by ward staff on admission or through existing staff communication channels. Participants self-selected to participate. Interviewees could return consent forms electronically or give verbal consent at the start of the interview, which was audio recorded and retained. Patient consent was not required as the research focused on the experiences of carers. The online survey for practitioners opened with the information sheet and consent statements and would only progress if participants actively indicated consent.

### Data collection

Interviews were conducted by telephone or using videoconferencing by a team of mental health social researchers, including two with experience of direct practice with unpaid carers and one with experience of social work practice within mental health services. Carers were interviewed about their experiences of leave, including communication, understanding, involvement and support around leave. Practitioners were asked about the use of the standard in a ‘live’ practice context to ensure a triangulated perspective. A series of core questions were asked with scope for participants to raise further issues they deemed important^22^ (Supplements 1 and 2 available at https://doi.org/10.1192/bjo.2025.16). Interviews were held between April 2022 and May 2023. However, recruitment for practitioners was low, with site feedback attributing this to staff time and workloads, hence the survey (Supplement 3) was offered as an alternative (open March–April 2023). The survey used open-ended questions based on the interview topic guide: the extent of use of the s.17 standard; benefits to using the standard; challenges and barriers; and scope for improvement. This allowed practitioners to participate at their convenience and was anticipated to reduce social desirability bias, thus resulting in more honest responses,^23^ albeit acknowledging the corresponding potential for misunderstanding of questions.^24^

### Data analysis

Responses to open-ended survey questions were analysed using content analysis.^22^ This analysis emphasised key concepts, reflecting the potential for analysis to lack sufficient qualitative depth if only key words are used.^25^

Interview data were coded, re-coded and analysed using a reflexive thematic analysis approach,^26^ with the coding frame refined iteratively following the re-reading of transcripts at each round of coding. Codes were analysed across transcripts to identify common themes in the data. A sample of transcripts were also coded by a co-author to check for robustness and consistency. The potential for conscious or unconscious bias in the design and framing of the survey and interview questions and the interpretation and communication of responses^27^ was addressed as far as possible through discussion and challenge among the research team and externally with the research advisory group, which included both practitioner representatives and unpaid carers.

### Ethics

All participants were actively informed verbally and in writing that involvement was voluntary and confidential. In-patient staff were blinded to carer recruitment and staff could participate anonymously using the online survey. All carers and those staff who completed an interview/survey outside working hours were sent a £20 shopping voucher as a thank you, with contact information separated from their data. All procedures contributing to this work comply with the ethical standards of the relevant national and institutional committees and with the principles of the Helsinki Declaration of 1975, as revised in 2008, in so far as these are relevant to non-medical research. Ethical approval for all procedures was granted by a NHS Research Ethics Committee (Ref: 21/WS/0156).

## Results

### Participants

Thirteen carers were recruited from five participating NHS trusts: seven from usual care wards and six from implementation wards. Of these, 11 took part in s.17 leave (six from usual care wards and five from implementation wards). Thirteen practitioners, with experience ranging from 5 months to 27 years, were also recruited from four trusts. Three were interviewed, while ten completed the survey. Sociodemographic characteristics for all participants are provided in Table 1.

**Table 1:** Sociodemographic characteristics of carer and practitioner participants

	Carers (*n* = 13)	Practitioners (*n* = 13)
Age		
18–24		2
25–39		5
40–54	4	3
55–69	4	3
70+	2	
Not disclosed	3	
Gender		
Male	4	3
Female	9	10
Ethnicity		
White	7	9
Asian	1	2
Black	1	
Mixed	1	1
Not disclosed	3	1
Role		
Parent	7	
Partner	3	
Sibling	2	
Son/daughter	1	
Medical		1
Nursing		5
Occupational therapy		1
Psychology		1
General healthcare		4
Not disclosed		1
Qualification status		
Role requiring formal qualification		6
Role not requiring formal qualification		6
Not disclosed		1

### The s.17 standard experience

Carers interacted with ward staff across three domains covered by the guidelines: information sharing (the extent to which carers were informed about the ward, the support available and the role of s.17 leave), decision-making (how carers were involved in planning s.17 leave and any changes to leave, including how decisions were made and communicated) and action-taking (how the ward prepared carers for s.17 leave, including the handover of care, referrals to support and any requisite follow-up).

**Fig. 2:**
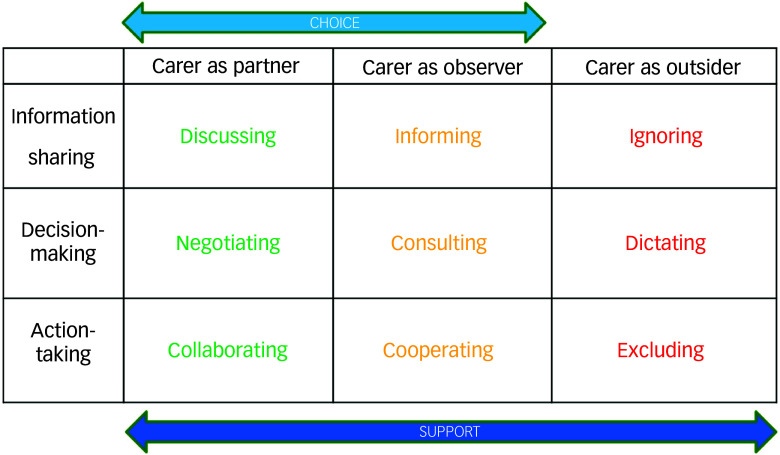
The carers’ partner–observer–outsider matrix.

Carers’ experiences of each domain varied widely. While the s.17 standard sought to position carers as equal partners in care, carers’ accounts positioned them sometimes as partners, but also as observers or outsiders, with each carer experiencing multiple levels of involvement around different aspects of their interactions with ward staff (Fig. 2).

Practitioners’ experience of and engagement with the s.17 standard demonstrated a similar level of inconsistency. Only five of the 13 practitioners reported attending training in the standard, and engagement with the practice activities varied considerably (Table 2).

**Table 2:** Intervention ward practitioner survey participants’ self-reported adherence to the section 17 (s.17) standard (participants are differentiated by trust (T1–T4) but not by ward to ensure anonymity, *n* = 10)

	T1	T2	T2	T3	T3	T1	T4	T2	T2	T2	Total
Attend regular training about s.17 leave and the role carers have in this	X										1
Speak with carers about any need they have for support	X	X	X	X	X		X		X	X	8
Refer carers for a carer assessment	X	X			X		X				4
Give carers information about life on the ward, including planned activities	X	X	X	X	X		X		X	X	8
Provide written information for carers about what s.17 leave is	X	X					X		X		4
Involve carers in planning for s.17 leave in advance	X	X			X		X		X	X	6
Involve carers in any changes to planned s.17 leave	X	X	X		X		X		X	X	7
Update carers on how the patient is at the start of s.17 leave	X	X	X	X	X		X		X	X	8
Make sure that carers know who to contact in case s.17 leave does not go well	X	X	X		X		X		X	X	7
Seek feedback from carers in private (away from the patient) after s.17 leave	X	X		X	X		X		X	X	7
Offer support to carers after s.17 leave that did not go well	X	X	X	X	X		X				6
Total	11	10	6	5	9	0	10	0	8	7	

#### 1. Partners in care

Carers experienced partnership as active involvement and participation in planning and enacting s.17 leave. Across both implementation and usual care wards, this happened most commonly in relation to facilitative action. This included practical organisation, a considerate approach to staff-escorted leave that enabled more normal family interactions and an educational approach from the ward on how to effectively support the patient during leave to promote recovery:

‘When she first came out we were very much like, wouldn’t let her go to more than like the toilet on her own kind of thing, “are you going to be okay?” […] the psychologist was wonderful. She had quite a few sessions with me, my dad and my mum as family ones. They really helped us to then use the leave as, this is normal life mate.’ (Implementation Carer 5 (IC5))

However, with information sharing, the partnership work was more one-sided. While carers reported some proactive approaches from wards to gather feedback following s.17 leave, this was not always accompanied by provision of relevant information beforehand, increasing the uncertainty around leave, as this carer explained:

‘I actually had to ask […] “do I need to do anything, you know, is there a number he needs to contact?” So I had to ask, I was being more proactive than somebody else saying “okay, come and sit here, we need to tell you about this and that”. They did eventually when I’d asked them a series of questions, like “do you have a phone number in case of emergencies, you know, is there anything I need to do?” Then I think, yeah, somebody else was called over and they said “oh, yeah, we just need to tell you about his medication” and, you know, seriously, they didn’t even tell me about what he needs to do, they just said “oh, he’s got his medication that he needs to take with him” which they needed to put in a bag, and I said “oh, can you please just go through it with me, so I know what’s going on in case he doesn’t take it”.’ (Usual Care Carer 1 (CC1))

Practitioners’ accounts suggested that such differences were embedded within the wards’ systems. Even within the same trust, variation could be substantial; seeking feedback could range from ‘agree[ing] a time to call them [carer] once they get home or for them to call the ward and give more in-depth feedback’ (Implementation Practitioner 2 (IP2)) to ‘we don’t intervene really, unless they ask us to’ (IP1). While both practitioners here described a process for checking in with carers following s.17 leave, the expectations were expressed very differently, with one ward acting proactively, and the other reactively. While ostensibly both wards sought feedback following leave, in line with the s.17 standard, the different approaches suggest substantial differences in experience.

Notably, carers’ positive experiences as partners in decision-making were often described as self-initiated, usually in response to dissatisfaction with progress towards or plans for s.17 leave and linked to access to consultants. However, this access, and therefore discussions that carers found more productive, was not always achieved without challenge, as one carer highlighted:

‘I had a very, you know, useful discussion with the consultant who, you know, was actually quite flexible. I mean, when the nurse phoned it made it sound as though, you know, this had been arranged and we’d just got to go along with it. But, you know, when I challenged that and asked to speak to the consultant, I mean the consultant was much more flexible and accommodating.’ (IC1)

Practitioners did not position themselves as responsible for interactions with carers. While there was an awareness that ‘it is beneficial to add carers in planning s.17 leave’ (IP10), this was frequently articulated as benefits for patients and for staff in terms of ‘easier discharge’ (IP11), ‘to see what is best for the patient’ (IP6) or ‘gaining information about how service users present’ (IP12). This reflected a recurring narrative that emphasised the patient as the ward staff’s priority, with time devoted to working with carers seen as ‘time taking staff off the ward’ (IP9). Carer interaction was suggested as a role for a ‘liaison person’ (IP3), which allowed for more consistent working with carers and better continuity of care, framing the engagement of multiple staff as ‘interfering’ (IP3). However, one practitioner noted a risk to carers’ well-being if engagement with carers was not a universal staff responsibility:

‘I would never let anybody [carer] off the ward, and sometimes they are upset, because things haven’t gone well, so you should spend time asking what has happened, what has gone wrong, what can we do. I am going to be honest, that doesn’t happen with everybody. Some of my colleagues, just let them out.’ (IP1)

#### 2. Observers of care

Observation represented more passive engagement, with carers’ views on the ward’s plans checked retrospectively. For some carers, being ‘told what was possible’ (IC4) represented sufficient involvement in decision-making, although it was acknowledged that this could affect the carer’s relationship with the ward, and correspondingly with the patient, if plans were made and then subsequently revised if the carers were unable or unwilling to support them, particularly if this was raised in front of the patient:

‘And because they’re right beside you, it would be a bit stressful to then have to turn round and say, “I’m really not comfortable with that”. I mean, ‘cause, and that’s a let-down for them if they’d gotten their hopes up that they can be let out. And that’s probably stressful for both people.’ (IC3)

For one retired carer there was recognition that their personal circumstances allowed for them to be ‘a bit more flexible probably than another person would be’ (IC4) in accommodating this informational approach. However, for some carers, such an approach did not account adequately for external commitments and was adopted ‘without any consideration for what was going on at home or, you know, how we felt about that’ (IC1).

This passive positioning may have been rooted in practitioners’ perception of carers as external to care. Practitioners spoke of inviting carers into care, enabling them to ‘feel involved’ (IP10) rather than viewing them as active contributors to the patient’s ongoing support. By placing carers outside of care provision in this way, practitioners operated with an expectation that carer involvement would be more passive and in line with the ward’s intentions rather than the carer’s needs:

‘I think sort of families and carers, they take a bit of a backseat on this, they are led by us to some extent, as to what they, to how it works really. Most seem quite compliant in doing that. You occasionally get the odd ones that perhaps know a little bit more about s.17 and perhaps know a little bit more about their rights as a carer.’ (IP1)

The communication of pre-made decisions raised challenges for some carers in understanding the plan. For one carer, who lived a substantial distance from the ward, s.17 leave was incorporated into a previously planned ward visit. However, as the carer was unclear what s.17 leave involved, the fact that this was unsuitable did not become apparent until their arrival, illustrating the limitations of ward-led decision-making where shared understanding of the plan is lacking:

‘If anything I’d have gone over earlier and took her out for the day, but because the visit was booked they didn’t, like, change that, they just left the visit for five. I already knew about it, but they’d already rang, they only rang on the Thursday to say, they just said it was great that I had a visit and that we would sort it out, we would see how we went then, but again five o’clock in the middle of nowhere it was like, you didn’t have nowhere to go or nothing to do anyway at that time. So, we were a bit stuck really.’ (CC4)

Timing of communications was also a particular issue for carers. Information shared late in the planning was potentially incomplete, or structured around the ward’s expectations, rather than carers’ self-identified needs, without carers necessarily having an opportunity to ask any questions. Plans for s.17 leave were ‘sort of dropped on us a bit’ (IC4), ‘really stressful’ (IC3) or ‘just came out of the blue’ (CC1). While carers always either accommodated the short-notice plans, or agreed an appropriate change, the lack of early involvement was a source of stress.

Practitioners framed engagement with carers in the wider context of competing demands, primarily incompatible ward routines, with one practitioner noting that carers’ time on the ward clashed with staff handover, or legal responsibilities, particularly around consent to share information. For some practitioners, carer engagement was conditional on patient consent, which minimised more active partnership working. In this, some practitioners acknowledged a fear of ‘falling foul of something’ in the context of a ‘blaming culture’ (IP1).

#### 3. Outsiders to care

As outsiders, carers were excluded from processes relevant to s.17 leave: they lacked information, were excluded from ward meetings and had a sense of ‘being left’ to deal with s.17 leave for themselves. The majority of carers received no prior information about s.17 leave, with only two having written information provided. However, this information was not necessarily given in ways that carers could relate to or understand in the stressful context of hospital admission, as one carer explained:

‘I scanned over it but because I, they’d sent it to me, but at the time that they sent it to me, he was under Section 2, so, I didn’t think it really had anything to do with me until they started talking about it.’ (IC3)

Correspondingly, carers’ understanding of s.17 leave was relatively limited, and sourced from a range of places, including family, friends, the internet and the patient themselves. None of the carers had a clear idea of their own or the patients’ rights around s.17 leave and, for some, it was involvement in the study that raised their awareness of it. This sense of being uninformed continued even when s.17 leave became a tangible plan.

Practitioners held conflicting views on information sharing with carers, as the two examples below illustrate:

‘I think we also need to be more proactive in having these conversations informally rather than expecting carers to come to us with issues.’ (IP8)

‘Carers should regularly visit the ward to know about the ward and ask questions face to face with the nursing team.’ (IP10)

For these two practitioners, the onus for information sharing sat in different spheres: on ward staff to facilitate ongoing communication or on carers to proactively raise questions and ensure they had access to necessary information. Practitioners presented this carer-led expectation as a default. Carers were not seen as barred from information, rather ‘if somebody does come and *they want to talk to them*, they will talk’ [emphasis added] (IP3). This expectation of carer-led engagement in the context of carers’ expressed lack of knowledge about s.17 leave created the potential for shortfalls in communication, which for one practitioner highlighted a key concern:

‘It is the ones that come in first time that I worry about, the ones that come in on a first experience of in-patient services, they are the ones that are very frightened and worried, it is something new, unknown, they have heard the horror stories, they are the ones that we need to look after, they are the ones that should come up on a list of people and we say you know, it is not as bad as what they say, that could be a big thing really.’ (IP1)

Narratives of support for carers were inconsistent. While two carers were referred to external support services, these services were both quickly withdrawn for reasons unclear to the carers. This compounded issues around a lack of support and information from the wards themselves that risked carers feeling isolated and unsupported in the context of leave:

‘I did feel a bit lacking in that area about them sharing information about what to do. Because if you felt unsafe, I feel like the [carer’s] not going to know what to do. And they [ward staff] just say, when they just, you know, advise you to call the ward, it’s, kind of, well, yeah, but what’s going to happen then? What do you do?’ (IC3)

This lack of information and support could lead to negative outcomes. For one carer, a lack of guidance and information from the ward about the in-patient admission precluded them helping the patient understand their progress and led to threats of violence during leave. By leaving them unprepared for the patient’s questions, the carer described the ward actions as directly contributing to the patient’s violent response, as follows:

‘It is very difficult, because how they [the ward] are kind of adds to how [patient] is in the fact that he will make demands of me that if I can do it then I will do it, and if I can’t then I can’t […] even though he threatens to smash my head in while I’m there.’ (IC2)

Exclusion from the wider processes of in-patient care also contributed to carers’ sense of being outside the progression to and through s.17 leave. Carers shared experiences of being excluded from ward meetings, either through not being invited, or because of these occurring at inaccessible times. Even where carers were involved, this did not mean they had the opportunity to contribute to decision-making or had pre-warning that s.17 leave was an option, as this carer highlighted:

‘I didn’t know anything about this temporary leave, it was actually during the, you know, conversation of the face-to-face meeting that we had that they talked about discharge and it was whilst they were talking about the discharge that they put in “oh, I think, you know, it would be good for him to go home for a few days before we discharge him”.’ (CC1)

Across wards, carers communicated a sense of being ‘left in the dark a lot of times’ (CC6) or ‘really puzzled’ (IC1), unsure of the overall purpose of leave, beyond being a route to recovery or to meet an expressed request. For one participant, this was something that could have been addressed through ‘more staff-initiated feedback or staff-initiated updates’ (CC6), although it was caveated that how useful this would be was highly dependent on individual practitioners.

Practitioners positioned this exclusion as arising from challenges within the staff–carer relationship. While some of this reflected an overt level of disagreement between ward staff and carers around practical aspects of s.17 leave, such as ‘challenging the amount of leave’ (IP13) or being ‘unwilling to accept the problems’ (IP7), there was also an overall sense of carer input as problematic if carers were unwilling to challenge the patient, or because their contribution could detrimentally affect the patient’s relationship with the ward, with concern around ‘disagreement between the parties’ (IP6) that could ‘fracture the trust the service users [sic] has’ (IP5).

This positioned carers as the key barrier to implementing the s.17 standard, either because of an unwillingness or an inability to engage with what the ward could offer. While this was automatically critical, it did place responsibility for difficulties in implementation with carers rather than within the ward. Notably, some critiques of carers mirrored those that carers had made of the wards themselves, such as around ease of contact:

‘Carers often have other responsibilities, commonly young children and work meaning that they can struggle to attend meetings and answer their phone reliably in work hours.’ (IP8)

While carers demonstrated frustration with perceived shortfalls in their involvement with the in-patient wards, they were not unsympathetic to the challenges. Carers recognised that ward staff ‘have a lot on’ (CC4), have ‘got a lot to deal with’ (IC3) and an awareness that ‘there’s obviously things going on, on the ward’ (CC5), which required that ‘expectations were adjusted’ (IC5) in terms of their interactions with those staff. Nonetheless, carers still expressed feeling ‘isolated’ (CC6) and ‘left behind’ (IC3), with instances of carers across contexts articulating that ‘I don’t even think they consider us carers’ (CC5).

Practitioners also acknowledged the practical challenges around engagement relating to the resources at their disposal, with limited staffing and time demands having an impact across their work. These resourcing issues were multifaceted and widespread, potentially leading staff to ‘prioritise the immediate tasks on the ward, like medication’ (IP2) in lieu of a more holistic approach. There was a combination of the practical need to prioritise patient care but also the indication that staff ‘may not feel they are responsible’ (IP9) for carer involvement, linked to earlier themes around carer externalisation and the staff–carer dynamic. Training was seen as welcome; however, there was also a keen awareness of a lack of opportunity for such training in the context of a practice environment where ‘half of the time you haven’t even got time to do your mandatory training never mind extra training’ (IP3).

## Discussion

### The overall impact of the s.17 standard

This study sought to explore comparable and subjective experiences of the use of the s.17 standard in practice. However, the standard was not consistently implemented and both carers and practitioners reported wide variety and inconsistency in use, which suggested that in-patient settings were unable to incorporate it into their usual practice. Instead, engaged and supportive practices were described alongside experiences of exclusion and avoidance regardless of implementation status. Levels of carer involvement were highly contextual, dependent on both individual practice settings and the involvement of specific practitioners, reflecting the findings from both the earlier phase of this study^17^ and previous research.^16,28^

Addressing such variation had been a key intention of the s.17 standard in introducing a framework to provide consistency in approach that could be replicated across practice contexts.^17^ By contrast to this intention, carers experienced a matrix of interactions as partners, observers and outsiders. While the s.17 standard sought to position carers within the ‘partnership’ range of interactions, carers’ and practitioners’ accounts more frequently placed them within the ‘outsider’ domain. However, carers experienced a wide range of interactions both individually and overall as a group.

Carers wanted a relationship with the ward that was supportive of their supporting the patient. Although previous review of the literature has suggested that carers value increased involvement,^29^ notably for some carers, involvement as an observer was seen as sufficient, letting the ward take the lead, particularly with regard to decision-making. However, this position was generally held by carers who had more flexibility and fewer external commitments, who could more easily accommodate plans devised on the ward. For those balancing external commitments or practical barriers, this position was experienced as more frustrating, indicating a need to engage with carers individually to establish suitable levels of involvement.

### The interaction of the s.17 standard and the practice environment

A consideration of systemic issues may help to explain the inconsistent implementation and variation in practice. Resource and staffing limitations are commonly identified as a barrier to intervention implementation,^30,31^ but in the context of a carer intervention this was compounded by practitioners’ belief that support for carers was not their primary role. This lack of role clarity and responsibility^6^ and a lack of confidence in working with carers,^17,19^ particularly in practice environments that were not supportive of carer engagement,^15,32^ were a barrier to both practitioner access to carers and carer access to practitioners.

Research has previously highlighted the value for carers in working directly with consultants^32^ and, notably, for some carers in this study, it was their interactions with consultants that best reflected the partnership engagement they sought. However, this may be reflective of power structures within mental health services rather than a differing professional approach. For detained patients, decision-making regarding leave from hospital is restricted to approved clinicians, a role overwhelmingly held by doctors.^33^ Where carers sought to be involved in decision-making, limited engagement from the wider ward teams may have represented a lack of power more than of will.

The findings indicated wider issues about the involvement of carers. Concerns around patient confidentiality and autonomy in relation to carer involvement as seen here are a longstanding source of tension.^15,20,32,34^ Such barriers can serve to discourage and limit carers’ involvement and restrict access to information.^34^ The s.17 standard explicitly excluded sharing patient information with carers, unless it related to s.17 leave in which the carer was involved. Nonetheless, concerns about appropriate information sharing persisted and affected carers’ experiences, perhaps reflective of perceptions or experiences of blame cultures within NHS contexts.^35^

Practitioners’ perception of carers also warrants consideration. Dirik et al^32^ described clinicians in their study as conceptualising carers in one of three context-driven roles also seen in this study: (a) useful resource; (b) troublemaker; or (c) invisible. Practitioners’ positioning of carers as the primary barrier to adherence to the s.17 standard both categorised carers as ‘troublemakers’ and was a potential rationale for exclusion (or invisibility), while the value of carers as a resource for patient care was the most common rationale for their inclusion. Although defining a carer in mental health contexts is challenging^32^ and carers did not always conceptualise themselves this way, recognition of and respect for their contribution as family or carer was both sought and expected.^6^

### Implications for practice

While the s.17 standard corresponded with what both carers and practitioners posited as ‘good practice’, low engagement with both the research and the implementation of the standard itself prevented any effective assessment of its efficacy in addressing the challenges carers experience around s.17 leave.^16,17^ Insufficient resourcing has ramifications for how effectively changes in practice can be adopted; however, these findings indicate a need both to consider how carers are perceived within mental health services and for clear guidelines on roles and responsibilities to support practitioners to engage carers in meaningful and effective ways. This may also require consideration at an organisational level of the role hospital admission plays in the wider context of a patient’s mental health experience.^32^

### Limitations

A small sample of carers and practitioners participated in this study. Although 13 participants is deemed sufficient to achieve code and theoretical saturation in qualitative research,^36,37^ an understanding of the relevant issues in full depth may have required additional interviews,^38^ and the extent to which participants are representative of their respective groups is difficult to gauge because of the self-selecting approach to recruitment and the use of gatekeepers to approach prospective participants. Nonetheless, the themes that were evident across geographical and practice settings are suggestive of recurring challenges that need consideration in terms of designing, testing and implementing interventions to support carers in health contexts. The study also notably took place during ongoing coronavirus pandemic restrictions, which affected carer interactions with some wards and may have influenced the experiences reported here.^39^ Further research is required to test whether full implementation of the s.17 standard improves outcomes for carers and, ultimately, the patients who they provide care for.

## Supporting information

Tucker et al. supplementary material 1 Tucker et al. supplementary material

Tucker et al. supplementary material 2 Tucker et al. supplementary material

Tucker et al. supplementary material 3 Tucker et al. supplementary material

## Data Availability

The data that support the findings of this study are available from the corresponding author, L.T., upon reasonable request.

## Supplementary material

The supplementary material for this article can be found at https://doi.org/10.1192/bjo.2025.16
