# Supplementary material for: Supporting unpaid carers during section 17 leave from mental health in-patient wards: carer and practitioner perspectives
Source: BJPsych Open. 2025 Mar 26;11(2):e71. doi: 10.1192/bjo.2025.16 (PMC12001960; doi:10.1192/bjo.2025.16)
Supplement: Tucker et al. supplementary material 1 — Tucker et al. supplementary material [file S205647242500016Xsup001.docx]

**Section 17 Leave: Supporting unpaid carers**

**Open-ended questions about s.17 leave**

***Section A: Information about s.17 leave***

1. What, if anything, do you know about section 17 leave? Where did you get this information from?
2. Were you provided with any written information about s.17 leave before the leave occurred? How helpful did you find this?

***Section B: Involvement in planning s.17 leave***

1. What involvement did you have in planning X’s first episode of s.17 leave?
2. In what ways, if any, would you like to have been further involved in planning for the s.17 leave?
3. Were you happy with when the leave took place and how much notice you had?
4. How might planning for s.17 leave be improved to better meet your needs?
5. Are you aware of what carers and patients can ask for in relation to s.17 leave?

***Section C: Support during the leave***

1. What, if any, information did the hospital share with you at the start of the leave, for example about X’s condition or current mood, care/support needs, places you might wish to take X?
2. Were you made aware that you could make requests for leave or ask for leave to be extended, etc?
3. Had you been informed what to do if you had any concerns during the s.17 leave?
4. Were any changes made to the leave and, if so, were you informed about these and agreed them in advance?
5. What could have made your experience of s.17 leave any better?
6. How much did the s.17 leave cost you (transport, entry fee for an activity, lunch, etc)? Who paid for this (carer, patient’s monies, hospital)? Was this manageable?

***Section D: Feedback following s.17 leave***

1. Did staff ask you for feedback on how the leave had gone or any issues you or X experienced during the leave? If so, was X present or were you asked in private?
2. If applicable, when and how were you asked for feedback? (e.g. in person immediately following the leave, by phone the next day, etc.)

***Section E: Carer support needs***

1. Did the ward staff talk to you about any support needs you may have as a carer prior to the s.17 leave taking place?
2. Do you feel that you have been provided with sufficient information about carer support services?
3. Have you been referred for a carers’ assessment?
4. To what extent did you feel supported by staff before, during and after s.17 leave?
5. In what ways, if at all, do you think you could be better supported by the inpatient staff?
6. In addition to what we have already discussed, are there any other ways in which the planning or experience of s.17 leave could be improved for you?
