# Supplementary material for: Supporting unpaid carers during section 17 leave from mental health in-patient wards: carer and practitioner perspectives
Source: BJPsych Open. 2025 Mar 26;11(2):e71. doi: 10.1192/bjo.2025.16 (PMC12001960; doi:10.1192/bjo.2025.16)
Supplement: Tucker et al. supplementary material 2 — Tucker et al. supplementary material [file S205647242500016Xsup002.docx]

**Section 17 Leave: Supporting unpaid carers**

**Topic guide for final practitioner interviews/workshops**

1. Can you tell me how use of the s.17 standard has changed over the past 12 months?
2. Looking back at the early teething problems identified during the previous round of interviews [go through teething problems previously identified in site/ward], to what extent have any of those issues been resolved or addressed? What difference, if any, did this make?
3. What has facilitated implementation of the s.17 standard?
4. What has hindered implementation of the s.17 standard? How, if at all, were those hindrances addressed?
5. To what extent are you following/implementing all 10 items in the s.17 standard? In addition to what you have told me already, what are the reasons behind not implementing all 10 items?
6. What, if any, benefits have you identified from using the s.17 standard? For staff? For carers? For patients?
7. What, if any, challenges have you identified from using the s.17 standard? For staff? For carers? For patients? How have those challenges been addressed?
8. Is the support offered to carers any different now to what it was prior to implementation of the s.17 standard? If so, how? How much time does this take compared to what you did before?
9. Has the provision of information leaflets for carers about s.17 had any impact on how staff discuss s.17 leave with carers or the questions or conversations that carers raise around s.17 leave? Does this take more time overall or has it saved staff time in other ways?
10. In what ways, if at all, has the information given to carers at the start of the s.17 leave changed? (e.g. are carers informed about care requirements, that they can request the leave is extended, etc). How much time does this take compared to what you did before?
11. How, if at all, has the introduction of the s.17 standard changed how/the extent to which practitioners involve carers in the planning of s.17 leave? How much time does this take compared to what you did before?
12. How, if at all, is feedback data collated and aggregated to inform future planning? How much time does this take compared to what you did before?
13. What, if any, further training, information or support do you think staff need to fully implement the s.17 standard?
14. What, if any, changes would you like to see to the s.17 standard, and why?
15. Is there anything else you would like to add?
