# Supplementary material for: Supporting unpaid carers during section 17 leave from mental health in-patient wards: carer and practitioner perspectives
Source: BJPsych Open. 2025 Mar 26;11(2):e71. doi: 10.1192/bjo.2025.16 (PMC12001960; doi:10.1192/bjo.2025.16)
Supplement: Tucker et al. supplementary material 3 — Tucker et al. supplementary material [file S205647242500016Xsup003.docx]

**Section 17 Leave: Supporting unpaid carers**

**Practitioner Survey**

***Survey Questions:***

***The s.17 leave standard (carers):***

*This section asks about your experience with the s.17 leave carers standard which was put into practice on your ward last year. You can still answer the questions even if you are unsure about the s.17 leave carers standard.*

Did you receive any training on the s.17 leave standard (either in person, via video call or using a recording)?

Yes  No  Can’t remember

Which, if any, of the following activities from the s.17 leave standard have you regularly done as part of your work over the last 6-12 months (please select all that apply)?

Attend regular training about s.17 leave and the role carers have in this

Speak with carers about any need they have for support

Refer carers for a carers assessment

Give carers information about life on the ward, including planned activities

Provide written information for carers about what s.17 leave is

Involve carers in planning for s.17 leave in advance

Involve carers in any changes to planned s.17 leave

Update carers on how the patient is at the start of s.17 leave

Make sure that carers know who to contact in case s.17 leave does not go well

Seek feedback from carers in private (away from the patient) after s.17 leave

Offer support to carers after s.17 leave which did not go well

*For the next few questions, please think about the activities on this list*

What benefits have you seen from working with carers in this way? If you have not been able to work with carers like this, what benefits would you predict based on your knowledge of working on your ward?

What downsides have you seen from working with carers in this way? If you have not been able to work with carers like this, what downsides would you predict based on your knowledge of working on your ward?

What challenges or barriers have you experienced or would you predict that make it difficult to work with carers in this way?

What other activities, if any, would improve how ward staff can work with carers in planning and undertaking s.17 leave?

Would you like to add anything else about working with carers around planning and undertaking s.17 leave?
